# Characterization of Subtype Selective Cannabinoid CB_2_ Receptor Agonists as Potential Anti-Inflammatory Agents

**DOI:** 10.3390/ph14040378

**Published:** 2021-04-19

**Authors:** Yaliang Tang, Barbara Wolk, Ryan Nolan, Caitlin E. Scott, Debra A. Kendall

**Affiliations:** Department of Pharmaceutical Sciences, University of Connecticut, Storrs, CT 06269, USA; Yaliang.Tang@UConn.edu (Y.T.); barbara.wolk@uconn.edu (B.W.); ryan.nolan@uconn.edu (R.N.); scottc@hendrix.edu (C.E.S.)

**Keywords:** cannabinoid CB_2_ receptor, agonists, inflammation, cytokines, cell migration

## Abstract

Activation of the CB_2_ receptor has been shown to have anti-inflammatory and antinociceptive effects without causing psychoactive effects. Previously, we reported that the compound ethyl 2(2-(*N*-(2,3-dimethylphenyl) phenylsulfonamido)acetamido)benzoate (ABK5) is a CB_2_ subtype selective agonist with anti-inflammatory and antinociceptive effects. In the present study, we tested four ABK5 derivatives, ABK5-1, ABK5-2, ABK5-5, and ABK5-6, to analyze the structure of ABK5 to obtain CB_2_-selective agonists with higher affinity and efficacy. Affinity, subtype selectivity, and G-protein coupling were determined by radioligand binding assays. Selected compounds were then subjected to evaluation of anti-inflammatory effects using two different cell lines, Jurkat (ABK5-1 and 5-2) and BV-2 cells (ABK5-1), which are models of T cells and microglia, respectively. ABK5-1, ABK5-2, and ABK5-6 had comparable CB_2_ binding affinity with ABK5 (and stimulated G-protein coupling), while only ABK5-1 and ABK5-2 maintained CB_2_-subtype selectivity. ABK5-5 did not bind CB_2_ in the detectable range. RT-PCR and ELISA analysis showed that the two compounds also inhibit IL-2 and TNF-α production, and they were more efficacious than ABK5 in inhibiting TNF-α production. CXCL-12 mediated chemotaxis was also evaluated by the transwell migration assay, and both ABK5-1 and ABK5-2 inhibited chemotaxis with a stronger effect observed in ABK5-1. In the microglia cell line BV-2, ABK5-1 inhibited IL-1β and IL-6 production, which suggests this compound has anti-inflammatory effects through targeting multiple immune cells, and may be a candidate for treatment of inflammation.

## 1. Introduction

Cannabinoid receptors are G-protein coupled receptors (GPCR)s, which are primarily coupled with G_i/o_ proteins. There are two subtypes, CB_1_ and CB_2_. CB_1_ is one of the most abundant GPCRs in the central nervous system (CNS) and is considered to be involved in control of neurotransmitter release [1,2]. On the other hand, CB_2_ is mainly expressed in immune cells and involved in regulation of immune response [3,4]. Both CB_1_ and CB_2_ are targets of Δ^9^-tetrahydrocannabinol (THC), a main psychoactive component of cannabis. THC binds and activates these receptors to induce various effects such as anti-nociception and anti-inflammation. However, activation of CB_1_ may also cause some unwanted psychoactive effects due to the physiological role of CB_1_ in the CNS [2]. This is a key problem that limits the medical use of cannabis despite its beneficial effects. In contrast to CB_1_, activation of CB_2_ is not considered to cause psychoactive effects [5,6,7,8], which makes CB_2_ an attractive target for treatment of pain and inflammation.

Inflammation is a response of the body to promote removal of harmful factors such as pathogens and damaged cells, largely driven by immune cells. Although inflammation is a natural protective response, prolonged and excessive inflammation after the harmful factors are eliminated from the body can cause some diseases including chronic pain [9,10]. Chronic pain is a major public health problem in the United States, which affects about one in five adults [11]. Although medications are available for treatment of chronic pain, effective medication for severe pain is still limited and may cause serious side effects, as exemplified by opioids. Therefore, effective analgesics with fewer side effects are highly demanded.

Inflammatory pain and neuropathic pain are two major types of chronic pain. In both cases, inflammatory mediators released from immune cells in the periphery or the CNS cause pain sensitization, which is characterized by enhanced pain caused by noxious stimuli (hyperalgesia) or due to normally innocuous stimuli (allodynia) [9,10,12]. Therefore, reduction of inflammation is considered to be useful to ameliorate associated pain. Activation of CB_2_ in immune cells by endocannabinoids such as 2-arachidonoylglycerol and N-arachidonoylethanolamine or exogenous agonists such as THC, WIN55212-2, and CP55,940 are reported to decrease production of nitric oxide, interleukin (IL)-1β, IL-2, and tumor necrosis factor (TNF)-α from various immune cells including macrophages, microglia, and T cells [13,14,15,16,17,18,19]. In addition to the inhibitory effect on cytokine production, CB_2_ agonists also decreased immune cell migration [19,20,21,22], which is another indication of anti-inflammation.

Previously, we identified a CB_2_ subtype selective agonist, ABK5, which is a structurally distinct compound from plant-derived or synthetic cannabinoid receptor agonists such as THC and CP55,940 [23]. ABK5 is a strong CB_2_ binder with K_i_ value of 16 nM, while no CB_1_ binding was detected up to 10 μM [23]. We also demonstrated that this compound has anti-inflammatory effects through inhibition of proliferation, IL-2 and TNF-α production, and chemotaxis in human T cell lines [23]. Moreover, ABK5, showed an anti-inflammatory effect in inflammatory pain model rats [24].

Although ABK5 partially decreased the mechanical threshold by Complete Freund’s adjuvant in an inflammatory pain model in rats, it only has limited effects [24]. Given that ABK5 is a lead compound discovered from a high throughput screen [25], it may only have limited potency, efficacy, or duration of action. In the present study, we examined binding of four ABK5 derivatives to further understand which part of ABK5 is critical for CB_2_-subtype selectivity so that we can optimize the compound to increase potency and efficacy without losing CB_2_-subtype selectivity. Among these compounds, two compounds, ABK5-1 and ABK5-2, which did not lose CB_2_ selectivity, were selected to be examined for verification that they still induce G-protein coupling and have anti-inflammatory effects in a human T cell line (Jurkat cells); ABK5-1 was also examined in a murine microglial cell line (BV-2 cells).

## 2. Results

**Figure 1:**
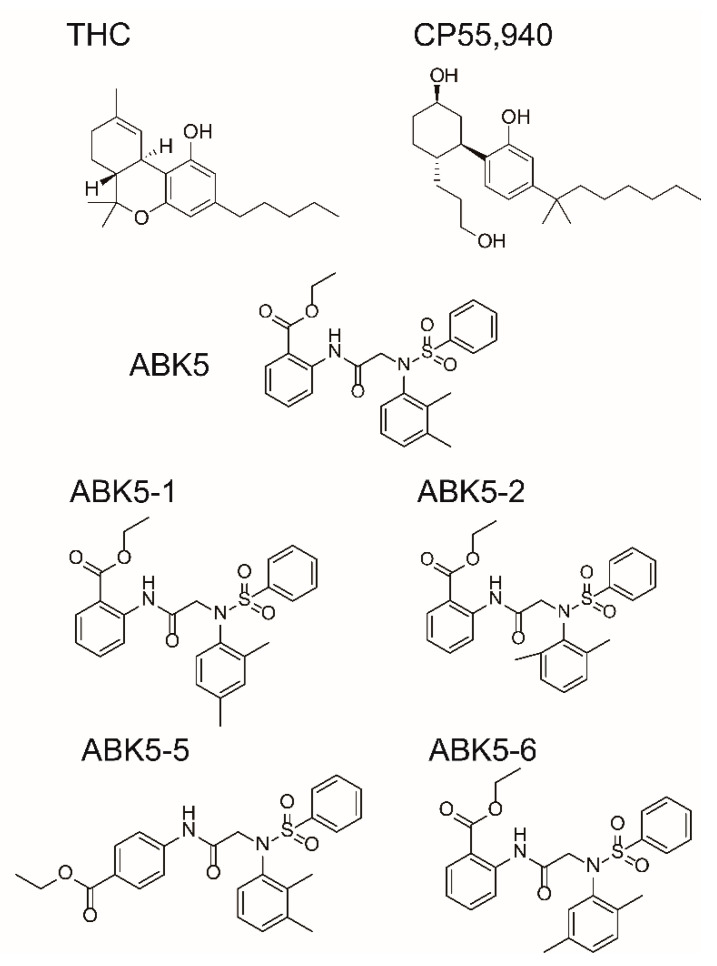
Chemical structures of some compounds that bind cannabinoid CB_1_ or CB_2_ receptors. Δ^9^-tetrahydrocannabinol (THC) and CP55,940 are agonists that bind both CB_1_ and CB_2_. ABK5-1, ABK5-2, ABK5-5, and ABK5-6 are examined in this study.

**Figure 2:**
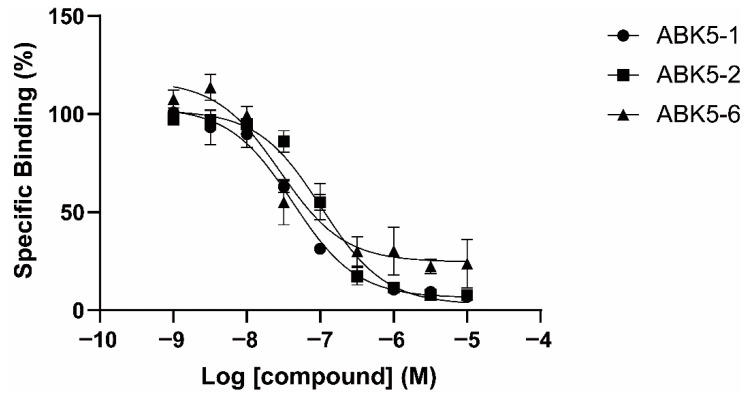
Specific binding of [^3^H]CP55,940 in the presence of increasing concentrations of ABK5-1 (circles), ABK5-2 (squares), ABK5-6 (triangles). Data are depicted as the mean ± S.D. of three independent experiments with each carried out in duplicate.

To evaluate binding affinity of ABK5 analogs that have a difference in the position of substituents in the *N*-phenyl ring (Figure 1), we performed competitive radiolabeled ligand binding assays using cell membrane from HEK293T cells transfected with human CB_2_ receptors. Figure 2 shows the specific binding of radiolabeled-cannabinoid receptor agonist [^3^H]CP55,940 to CB_2_ in the presence of various concentrations of test compound between 1 nM to 10 μM. ABK5-1, ABK5-2, and ABK5-6 dose-dependently competed with [^3^H]CP55,940 and decreased the specific binding of the radiolabeled agonist, which reflects the binding of test compounds to CB_2_. ABK5-1 bound to CB_2_ with K_i_ = 28 nM (Figure 2 circles). ABK5-2 (Figure 2 squares; K_i_ = 69 nM) had a marginally-lower affinity than ABK5-1 for CB_2_. ABK5-5 exhibited a highly unusual response to CB_2_ with little binding, and not exceeding 60% of total binding (data not shown), which made calculating the K_i_ value not useful. Therefore, ABK5-5 was not considered for further evaluation in this study. Finally, ABK5-6 bound CB_2_ most strongly among the four compounds (Figure 2 triangles; K_i_ = 14 nM). We also examined CB_2_ selectivity (versus CB_1_) of the three compounds by performing similar binding assays using membrane from CB_1_ expressing HEK293 cells instead of CB_2_ expressing cells. Neither ABK5-1 nor 5-2 bound the CB_1_ receptor up to 10 μM, ABK5-6, which bound CB_2_ strongly, had weak CB_1_ binding in the micromolar range. Therefore, ABK5-1 and 5-2 maintained the CB_2_ subtype selectivity of ABK5.

**Figure 3:**
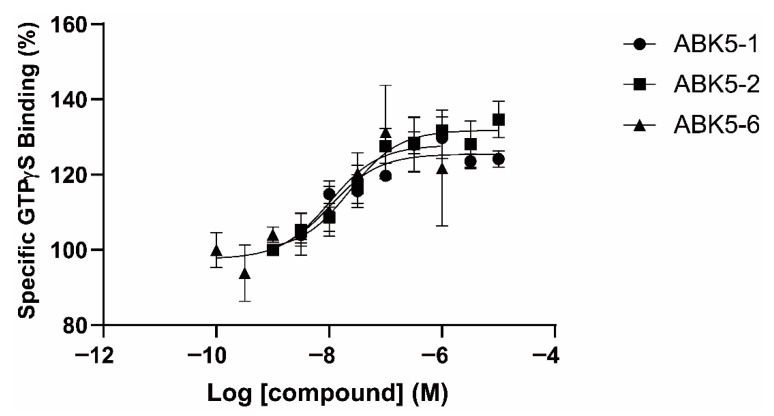
Specific [^35^S]GTPγS binding to CB_2_ induced by ABK5-1, ABK5-2, and ABK5-6. Concentrations of ABK5-1 (circles), ABK5-2 (squares), ABK5-6 (triangles) that caused stimulation of the specific binding of [^35^S]GTPγS to CB_2_. Data are depicted as the mean ± S.D. of three independent experiments with each experiment carried out in duplicate.

We also examined if the compounds can induce G-protein coupling and act as CB_2_ agonists. Three compounds, ABK5-1, ABK5-2, and ABK5-6, were subjected to [^35^S]GTPγS binding assays. Increasing concentrations, from 1 nM to 10 μM of the test compounds, were incubated with CB_2_ containing HEK293T cell membrane and [^35^S]GTPγS, which binds to G_i/o_ in the membrane upon CB_2_ activation and G-protein coupling. All three compounds stimulated dose-dependent increases of specific [^35^S]GTPγS binding (Figure 3). As expected from the binding affinity to the CB_2_ receptor, assuming full efficacy, ABK5-1 was more potent (Figure 3 circles; EC_50_ = 11 nM) than ABK5-2 (Figure 3 squares; EC_50_ = 24 nM). ABK5-6 had a similar potency as ABK5-1 (Figure 3 triangles; EC_50_ = 10 nM). These parameters are summarized in Table 1. These results suggested that, similar to their parent compound, ABK5, ABK5-1, and ABK5-2 are potent CB_2_-subtype selective agonists, which cause G-protein coupling.

**Figure 4:**
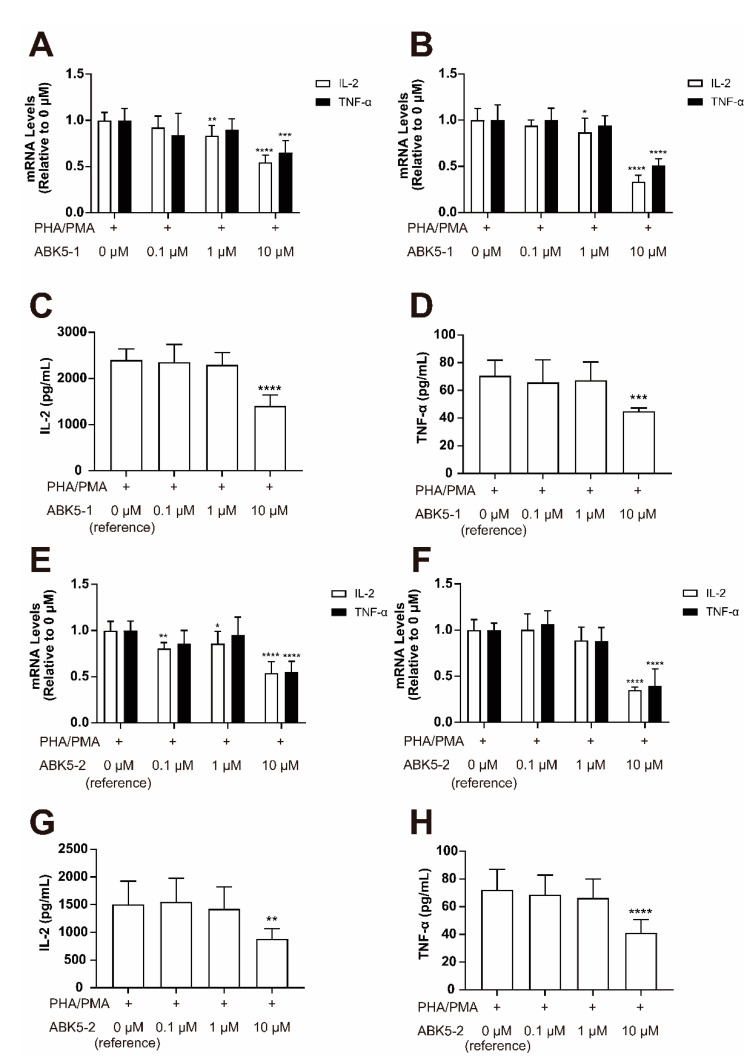
Effect of ABK5-1 and ABK5-2 on phytohemagglutinin (PHA)/phorbol 12-myristate 13-acetate (PMA)-induced cytokine production in T cells. Jurkat cells were pretreated with DMSO (<0.1%; indicated as 0 μM) or various concentrations of ABK5-1 (**A**–**D**) or ABK5-2 (**E**–**H**) and stimulated with PHA and PMA. IL-2 and TNF-α mRNA levels were determined by real-time PCR (RT-PCR) after ABK5-1 treatment and (**A**) 3 h and (**B**) 24 h of PHA/PMA (phytohemagglutinin/phorbol 12-myristate 13-acetate) stimulation. Protein levels of (**C**) IL-2 and (**D**) TNF-α were also examined after 24 h of stimulation. IL-2 and TNF-α mRNA were also examined after ABK5-2 treatment and (**E**) 3 h and (**F**) 24 h of stimulation, and protein levels of (**G**) IL-2 and (**H**) TNF-α were measured after 24 h stimulation. Results are indicated as the mean ± S.D. of three independent assays performed in triplicate for each concentration. One-way ANOVA and Dunnett’s post-hoc test were used and * *p <* 0.05, ** *p <* 0.01, *** *p <* 0.005, **** *p <* 0.0001 (versus reference).

Activation of CB_2_ is known to have anti-inflammatory effects. There are some studies reporting that CB_2_ agonists decrease cytokine production in immune cells [14,19,26]. We also reported that the cannabinoid receptor agonist CP55,940 and ABK5 inhibit cytokine production in the human T cell lines Jurkat and MOLT-4 [24]. Since ABK5-1 and 5-2 were confirmed to be CB_2_-subtype selective agonists, we were interested to determine whether these compounds also have anti-inflammatory effects. Note, although ABK5-6 had marginally better binding affinity to CB_2_, it bound to CB_1_ very, very weakly (i.e., micromolar range). Therefore ABK5-6 was not selected for further evaluations. We first tested the change of IL-2 and TNF-α mRNA levels in Jurkat cells. Three hours of ABK5-1 treatment resulted in a dose-dependent decrease of IL-2 mRNA with about 17% decrease at 1 μM and 46% decrease at 10 μM (Figure 4A, white columns). Similarly, TNF-α mRNA levels at 10 μM ABK5-1 were also significantly decreased by 35% (Figure 4A, black columns). This decrease of mRNA levels was also observed after 24 h of ABK5-1 treatment, which showed about 14 and 67% decrease of IL-2 in cells treated with 1 and 10 μM ABK5-1, respectively, and a 50% decrease of TNF-α in cells treated with 10 μM ABK5-1 (Figure 4B). To test if these mRNA changes are reflected in the protein levels of cytokines, the IL-2 and TNF-α concentration in the cell culture medium were measured by ELISA. Both IL-2 and TNF-α production significantly decreased by about 42 and 37% at 10 μM after 24 h of compound treatment (Figure 4C,D). Treatment of ABK5-2 also caused a decrease of mRNA and protein levels of the two cytokines. Unlike ABK5-1, at 3 h of treatment, the lowest concentration of ABK5-2 used caused the IL-2 mRNA decrease. IL-2 mRNA levels significantly decreased by about 20% at 0.1 μM, 15% at 1 μM, and 46% at 10 μM ABK5-2 (Figure 4E, white columns). On the other hand, TNF-α mRNA decreased by 45% at 10 μM ABK5-2 after 3 h of treatment (Figure 4E, black columns). At 24 h, only 10 μM ABK5-2 significantly decreased IL-2 mRNA (about 65% decrease; Figure 4F white columns) and TNF-α mRNA (about 60% decrease; Figure 4F black columns). These mRNA level changes were similar to that of ABK5-1, except ABK5-2 caused a stronger inhibitory effect on expression of TNF-α mRNA. For protein levels, a significant decrease of both IL-2 and TNF-α production was observed at 10 μM ABK5-2, which resulted in about 42 and 43% decrease of IL-2 and TNF-α, respectively (Figure 4G,H). Consistent with the mRNA levels, the inhibitory effect of ABK5-2 on TNF-α production was stronger than that of ABK5-1.

**Figure 5:**
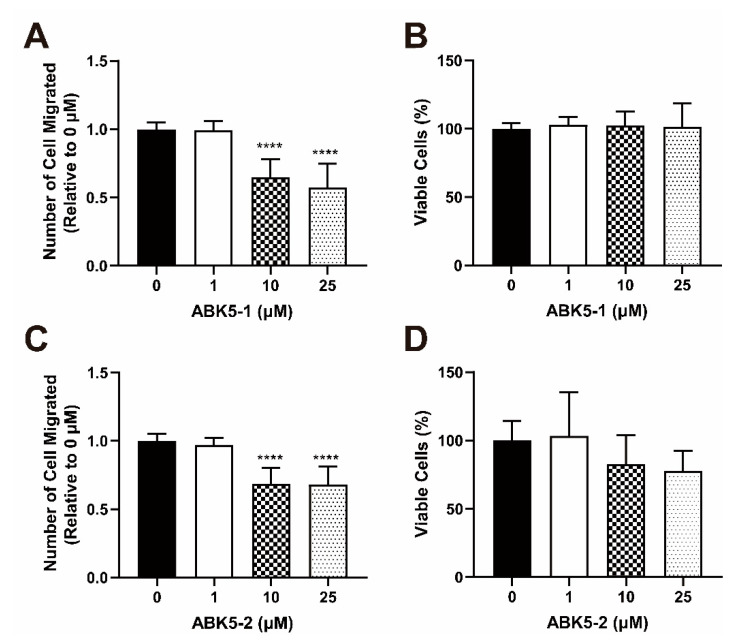
Effect of ABK5-1 and ABK5-2 on CXCL12-mediated Jurkat cell migration. Transwell migration of Jurkat cells treated with (**A**,**B**) ABK5-1 or (**C**,**D**) ABK5-2. Frames (**A**,**C**): Cells were treated with vehicle or various concentrations of test compounds for 2 h and then allowed to migrate through transwells for 2 h. Cell numbers migrated to the lower chamber are indicated as relative to vehicle (DMSO) alone. Results are indicated as the mean ± S.D. of three independent assays performed in triplicate for each concentration. Viability of cells treated with (**B**) ABK5-1 or (**D**) ABK5-2 after the migration assay was also examined. Cells were treated exactly the same but without transwells and viability was determined. Results are indicated as the mean ± S.D. of three independent assays performed for each concentration. One-way ANOVA plus Dunnett’s post-hoc test were used for both migration and viability assays. **** *p <* 0.0001 (versus vehicle control group).

During inflammation, immune cells are attracted by chemoattractants and migrate to the site of inflammation. Therefore, an inhibitory effect on chemotaxis of immune cells is also a sign of anti-inflammatory effect. We evaluated ABK5-1 and ABK5-2 to determine if these compounds can inhibit CXCL12-mediated Jurkat cell migration. Jurkat cells were incubated with various concentrations of ABK5-1 or 5-2 and loaded on the transwells and allowed to migrate through the transwells to the bottom chamber, which include CXCL12. The cell number in the bottom chamber was determined after 2 h of loading. ABK5-1 inhibited Jurkat cell migration at 10 and 25 μM (Figure 5A). ABK5-1 resulted in about 35 and 43% decrease of cell migration at 10 and 25 μM, respectively (Figure 5A). However, there was little change in cell viability with the corresponding treatment (Figure 5B). ABK5-2 treatment caused a 32% decrease of cell migration at both 10 and 25 μM (Figure 5C). Again, to verify these observations were not due to cytotoxicity of test compounds, cell viability was also monitored by incubating cells with compound and CXCL12 without the transwells. Although 10 and 25 μM ABK5-2 treatment resulted in a marginal decrease of cell viability (Figure 5D), which was not significant. This result suggested that some decrease of cell migration by ABK5-2 may have been affected by cytotoxicity of the compound, while the effect of ABK5-1 was only caused by inhibition of cell migration.

**Figure 6:**
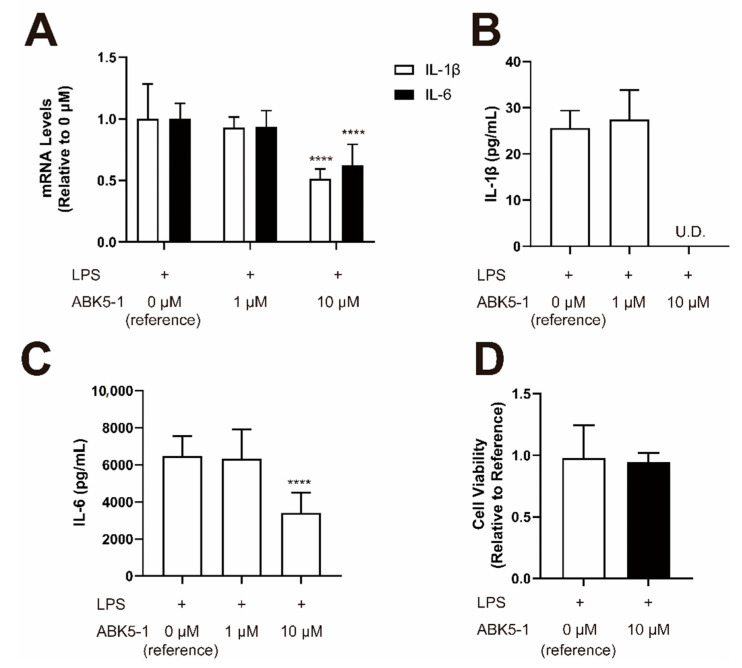
Effect of ABK5-1 on lipopolysaccharide (LPS)-induced cytokine production in microglia. BV-2 cells were pretreated with DMSO (<0.1%) or 1 or 10 μM ABK5-1 and stimulated with LPS. (**A**) mRNA levels of IL-1β and IL-6 after 6 h of LPS stimulation. (**B**) IL-1β protein concentration in the cell culture medium after 24 h of LPS stimulation. U.D. indicates under detection limit. (**C**) IL-6 protein concentration in the cell culture medium after 24 h of LPS stimulation. (**D**) Viability of BV-2 cells after ABK5-1 10 μM treatment. Results are indicated as the mean ± S.D. of three independent assays performed for each concentration. One-way ANOVA plus Dunnett’s post-hoc test were used and **** *p <* 0.0001 (versus reference).

To further evaluate the anti-inflammatory effects, we selected ABK5-1, which was the most potent, subtype selective, and did not affect cell viability, for testing the inhibitory effect on cytokine production in a different cell line, BV-2 cells. We stimulated BV-2 by lipopolysaccharide (LPS) and confirmed induction of pro-inflammatory cytokine IL-1β and IL-6 (data not shown). ABK5-1 significantly decreased mRNA levels of IL-1β by 63% and IL-6 mRNA by 48% at 10 μM (Figure 6A). These effects were also confirmed by cytokine protein levels. Also, 10 μM ABK5-1 greatly reduced IL-1β cytokine concentration in the culture medium to a level under the detection limit (Figure 6B). Similarly, the IL-6 cytokine concentration in the culture medium was also significantly decreased by 47% after treatment of 10 μM ABK5-1 (Figure 6C). ABK5-1 treatment did not affect the cell viability at 10 μM (Figure 6D). These results supported the possibility that ABK5-1 is an anti-inflammatory agent.

## 3. Discussion

Chronic pain and inflammation are closely related, with various immune cells involved in sensitization of pain in the periphery and the CNS. In normal conditions, mechanical, thermal, and chemical stimuli are detected by peripheral sensory neurons which are called nociceptors. Once the intensity of the stimuli reaches the threshold, the stimuli cause excitation of the nociceptors, and this signal is transmitted to the brain through second order neurons and other interneurons [10,12]. Inflammatory mediators and neurotropic factors released from immune cells activate their nociceptors and other second order and interneurons to increase the sensitivity of neurons [27]. This leads to hyperalgesia and allodynia [9,12]. Therefore, treatment to reduce inflammation is effective in reducing pain. 

Although studies reported that activation of CB_2_ can reduce pain through anti-inflammation [7,28,29], there is still no CB_2_-subtype selective agonist on the market. Previously, we identified a compound, ABK5, which was originally found to activate CB_2_ in a high throughput screening [25], and to be a CB_2_-subtype selective agonist [23]. ABK5 inhibited IL-2 and TNF-α production in Jurkat cells and MOLT-4 cells, and mitigated inflammation in a Complete Freund’s adjuvant treated inflammatory pain model in rats [24]. However, this compound had limited effects, which led us to undergo further structural analysis for future optimization. In the present study, we introduced four ABK5 derivatives to determine if we could improve ABK5 in CB_2_ binding and subtype selectivity. Moving the second methyl group from the 3-position in the *N*-phenyl ring to the 4, 5, or 6 position resulted in minor shifts in potency of CB_2_ binding. The 2,5-dimethyl compound, ABK5-6, showed a slight binding to CB_1_, while the other isomers were still subtype selective, up to 10 μM. On the other hand, change in the position of the benzoate from the 2 to the 4 position seemed to abolish CB_1_ and CB_2_ binding. Therefore, further modifications may be made on the N-phenyl ring to optimize chemical or biological properties such as solubility, stability, and metabolism of the compound.

Similar to ABK-5, we demonstrated that two derivatives, ABK5-1 and 5-2 activate the CB_2_ receptor by GTPγS binding assays. ABK5-1 had a similar EC_50_ for G-protein coupling compared with ABK5, while ABK5-2 had a weaker effect on inducing GTPγS binding. This was expected from the K_i_ values of two compounds provided they were equally efficacious. Consistent with prior studies showing the anti-inflammatory effects of CB_2_ agonists [13,14,15,16,17,18,30], both compounds inhibited PHA/PMA-induced IL-2 and TNF-α production. Although K_i_ and EC_50_ values calculated from binding and GTPγS binding were different than the concentration used for treating cells, which is common when performing functional evaluation using immune cells [16,20,31,32,33,34]. This is not surprising considering Ki values were calculated from assays in a simple environment only including the cell membrane and compounds in glass tubes, compared with functional assays using live cells. Test compounds can be metabolized, degraded, or adsorbed to plastic used in cell culture. Interestingly, ABK5-2 seemed to have a stronger effect in inhibiting TNF-α production than ABK5 (33% decrease in mRNA and 30% decrease in protein level) [24] and ABK5-1, despite having a lower affinity and potency than these compounds. After 3 h of compound treatment, 0.1 μM ABK5-2 significantly decreased IL-2 mRNA, while the lowest concentration of ABK5-1 did not show a significant decrease of IL-2 mRNA. ABK5-2 may have higher efficacy in reducing inflammation than ABK5-1. ABK5-2 mediated a decrease of IL-2 mRNA only at 10 μM after 24 h treatment. ABK5-1 still showed the effect at 1 μM after 24 h treatment. This may be due to a different stability of the compounds in cell culture media or metabolism by cells. 

Activation of CB_2_ causes the cellular response through various signaling pathways, including a change of adenylate cyclase activity and the cAMP concentration in the cells. Anti-inflammatory effects of CB_2_ agonists in T cells seems to be G-protein-dependent, and changes of the cAMP concentration activates protein kinase A, which inhibits T cell receptor signaling [14]. Since ABK5 derivatives also cause G-protein coupling, their anti-inflammatory effects may also be mediated by a similar mechanism. Future studies will include analysis of the potential mechanism of anti-inflammatory effects of ABK5 derivatives.

Although not significant, ABK5-2 showed a tendency of reducing cell viability at a concentration higher than 10 μM. This may contribute to the observation that ABK5-2 had a stronger effect on inhibition of cytokine production. However, this issue on cytotoxicity can be cell type specific. Some CB_2_ agonists are reported to have anticancer effects and induce apoptosis of cancer cell lines [31,35,36]. Considering Jurkat cells are frequently used since they are immune cells, but is also a cell line derived from leukemia, this cytotoxicity may be reflecting the anticancer effect of ABK5-2. It would be interesting to test and compare effects of ABK5-1 and ABK5-2 using non-cancer derived immune cells such as peripheral blood mononuclear cells (PBMCs) in the future.

Microglia are macrophage-like cells in the CNS, and activation of microglia is associated with chronic pain, especially neuropathic pain [37]. Damage of peripheral nerves causes release of mediators such as the chemokine CCL2, glycoprotein CSF1 and adenosine triphosphate from the central termini of nociceptors [37,38]. These mediators activate microglia, which in turn secrete pro-inflammatory cytokines and neurotropic factors [38]. Similar to the pro-inflammatory cytokines released from peripheral immune cells, these cytokines and neurotropic factors cause pain sensitization. Therefore, anti-inflammatory agents are considered to be effective for inflammatory pain, and to be useful for treating neuropathic pain. CB_2_ is expressed in microglia, and the level of expression is further enhanced when the microglia are activated [39,40,41]. Both endocannabinoids and exogenous cannabinoid receptor agonists are reported to decrease production of pro-inflammatory cytokines in primary microglia and microglial cell lines [19,34,42,43]. In agreement with these studies, our results also showed inhibitory effects of a CB_2_ agonist ABK5-1 on inhibiting production of IL-1β and IL-6, supporting the beneficial effects of CB_2_ in inhibition of neuroinflammation. In addition, microglia are also known to be able to produce anti-inflammatory cytokine IL-10 when they undergo alternative activation [44]. Cannabinoid receptor activation by endocannabinoids and some synthetic agonists are also reported to increase IL-10-producing anti-inflammatory microglia (commonly known as M2 microglia) [34,42,43,45,46]. In contrast, CB_2_ activation is also reported to promote microglia activation to pro-inflammatory (known as M1 microglia) phases in cancer pain with morphine tolerance [47]. It seems that CB_2_ agonists have different effects regarding polarization of microglia depending on the disease, but overall have beneficial effects. Since our results showed that ABK5-1 decreased IL-1β and IL-6, which are pro-inflammatory cytokines typically produced by M1 microglia, to similar levels, we hypothesize ABK5-1 decreases M1 microglia rather than simply affecting signaling pathways regulating production of each cytokine. Still, it will be interesting to examine how ABK5-1 affects the activation state of microglia in different models including both cellular and animal models, and future studies will include evaluation of a broader range of cytokines and activation markers after compound treatment to understand the impact of ABK5-related compounds on microglia activation.

Since the evaluations in the present study were performed using cell lines, there are limitations in some aspects of anti-inflammation such as types of cytokines the cells produce and cell viability. Testing the compounds in primary cells (e.g., PBMCs and primary microglia) will be helpful to further characterize the anti-inflammatory property of the compounds by globally analyzing change of cytokines and evaluating cell proliferation. In addition, it will be interesting to test anti-inflammatory and anti-nociceptive effects of compounds in inflammatory and neuropathic pain animal models in the future.

## 4. Materials and Methods

### 4.1. Reagents

ABK5-1 was purchased from ChemBridge Corporation (San Diego, CA, USA) and ABK5-2 was from Enamine (Monmouth Junction, NJ, USA). [^3^H]CP,55940 and [^35^S]GTPγS were purchased from PerkinElmer life Sciences (Waltham, MA, USA). CLCX12 was obtained from R&D Systems (Minneapolis, MN, USA). Phytohemagglutinin (PHA) and phorbol 12-myristate 13-acetate (PMA) were purchased from Millipore Sigma (St. Louis, MO, USA). 

### 4.2. Cell Culture

HEK293T cells, used for ligand binding and GTPγS experiments, were cultured in Dulbecco’s Modified Eagle Medium (Life Technologies, Carlsbad, CA, USA) supplemented with 10% fetal bovine serum (FBS), 4.5 mg/mL D-glucose. Jurkat cells, used for cytokine release and migration analysis and shown in Figure 4 and Figure 5, were cultured in Roswell Park Memorial Institute (RPMI) 1640 medium supplemented with 10% FBS and 1% antibiotic-antimycotic (Life Technologies, Carlsbad, CA, USA). BV-2 cells, used for cytokine release analysis as shown in Figure 6, were cultured in Minimum Essential Medium (Life Technologies, Carlsbad, CA, USA) supplemented with 10% FBS. All three types of cells were maintained at 37 °C and 5% CO_2_ saturation.

### 4.3. Cannabinoid Receptor Expression and Membrane Preparation

In the experiment, 1 × 10^6^ HEK293T cells were seeded in a 10-cm cell culture dish and cultured for 24 h. Cells were transfected with CB_1_ or CB_2_ using the calcium phosphate method. Approximately one day after transfection, cells were harvested and centrifuged at 4 °C for 5 min at 500× *g*. The pellet was washed with phosphate buffered saline (PBS) twice and resuspended in PBS containing 1% protease-inhibitor cocktail (Sigma-Aldrich, St. Louis, MO, USA) consisting of 104 mM 4-(2-aminoethyl) benzene sulfonyl fluoride hydrochloride (AEBSF), 80 μM apronitin, 4 mM bestatin, 1.64 mM E-64, 2 mM leupeptin, 1.5 mM pepstatin A. Then the cells were subjected to nitrogen cavitation under 750 psi for 5 min using a Parr cell disruption bomb. The lysate was centrifuged at 500× *g* for 10 min at 4 °C and the supernatant was spun at 116,000× *g* for 45 min at 4 °C. The pellet, consisting of the cell membrane expressing CB_1_ or CB_2_, was resuspended in TME buffer solution (25 mM Tris-HCl, 5 mM MgCl_2_, and 1 mM EDTA, pH 7.4) with 7% (*w*/*v*) sucrose/TME buffer. The concentration of the membrane protein was determined by the Bradford assay (Bradford, 1976).

### 4.4. Ligand Binding

Membrane protein was mixed with 1 nM to 10 μM of the test compounds and 1 nM [^3^H]CP55,940 (164.9 Ci/mmol; PerkinElmer life Sciences, Waltham, MA, USA), and TME buffer supplemented with 0.2% BSA was used to bring the total volume to 200 μL. [^3^H]CP55,940 acts as tracer and agonists that bind the target receptor compete with [^3^H]CP55,940. Samples were incubated for 1 h at 30 °C. Ten μM unlabeled CP55,940 was used to determine non-specific binding. Reactions were stopped by adding 5% BSA/TME buffer (*w*/*v*) and samples were filtered through a Brandel cell harvester using Whatman GF/C filter paper. Ultima Gold XR liquid scintillation cocktail (PerkinElmer Life Sciences, Waltham, MA, USA) was then added and vortexed. A Beckman Coulter 6500 liquid scintillation counter was used to measure the radioactivity of the samples.

### 4.5. GTPγS Binding

GTPγS binding assay buffer (50 nM TRIS-HCl, pH 7.4, 3 mM MgCl_2_, 0.2 mM EGTA, and 100 mM NaCl) membrane protein, 1 nM to 10 μM of the test compounds, 0.1 nM [^35^S] GTPγS (1250 Ci/mmol; PerkinElmer Life Sciences, Waltham, MA, USA), 0.1% fatty acid free BSA (*w/v*), 10 μM guanosine diphosphate (GDP) and GTPγS was incubated for 1 h at 30 °C with shaking. Ten μM unlabeled GTPγS was used to determine non-specific binding. Reactions were terminated by filtration with a Brandel cell harvester trapping the bound compounds in Whatman GF/C filter paper. A Beckman Coulter 6500 liquid scintillation counter was used to measure the radioactivity of the samples.

### 4.6. Compound Treatment for Cytokine Analysis

For cytokine analysis, 0.75 × 10^6^ Jurkat cells per well were seeded in 12-well cell culture plates and treated with compounds at a concentration between 0.1 to 10 μM and incubated for 30 min at 37 °C. Then cells were stimulated for cytokine production with PHA (1 μg/mL) and PMA (12.5 ng/mL), for 3 and 24 h. After stimulation, cells were harvested for RNA analysis and the culture supernatant was used for measurement of IL-2 and TNF-α concentrations by human IL-2 and TNF-α ELISA kits (Thermo Fisher Scientific; Waltham, MA, USA) according to the manufacturer instructions.

### 4.7. Quantitative Real-Time PCR

Jurkat cells were lysed and total RNA was extracted using TRIzol Reagent (Life Technologies, Carlsbad, CA, USA) followed by reverse transcription by a High-Capacity cDNA Reverse Transcription kit (Life Technologies, Carlsbad, CA, USA) according to the manufacturer’s instructions. Quantitative real-time PCR was performed using an Applied Biosystems 7500 Fast Real-Time PCR system in a 10 μL reaction volume containing 2 μL diluted cDNA and 0.5 μM each of forward and reverse primers, and PowerUp SYBR Green Master Mix (Life Technologies, Carlsbad, CA, USA). Primers used in the reaction were as follows: human 36B4 forward, 5′-GAAGTGCTTGATATCACAGAGGAA-3′, and human 36B4 reverse, 5′-TGATGCAACAGTTGGGTAGC-3′, and human IL-2 forward, 5′-AAGTTTTACATGCCCAAGAAGG-3′, and human IL-2 reverse, 5′-AGTCCCTGGGTCTTAAGTGAAA-3′, and human TNF-α forward, 5′-CAGCCTCTTCTCCTTCCTGAT′, human TNF-α reverse, 5′-GCCAGAGGGCTGATTAGAGA-3′. The PCR cycles are as follows: 50 °C for 2 min, 95 °C for 2 min, 40 cycles of 95 °C for 3 sec and 60 °C for 30 s.

### 4.8. Migration Assay

The migration assay was performed as described previously [24]. Briefly, a Transwell Multiple Well Plate with permeable polycarbonate membrane inserts (0.5 μm, 24-well, Corning, Corning, NY, USA) were used in migration assays. Jurkat cells were seeded at 2.5 × 10^6^ cells/mL in RPMI medium containing 2.5% FBS and treated with DMSO or test compound for 2 h. Then a sample of cells were transferred to the top well. RPMI medium containing 0 to 1000 ng/mL CXCL12 and the appropriate concentration of test compound was loaded to the bottom chamber. Cells were allowed to migrate for 2 h and cells migrated from the top well to the bottom chamber were counted or measured by CCK-8 (Dojindo Molecular Technologies, Inc., Rockville, MD, USA) following the manufacturer instructions. For evaluation of cell viability, the same procedure used for the migration assay was performed without the transwells and the viable cells were counted or measured by CCK-8.

### 4.9. Data Analysis

Results are presented as the mean ± standard deviation (S.D.). The ligand binding assays and the GTPγS binding assays were both carried out in duplicate for three independent experiments. The K_i_ values in the ligand binding assay were calculated using the Prism software (GraphPad Software Inc., San Diego, CA, USA), using IC_50_ values obtained from non-linear regression fitted to the one-site binding model. The Cheng-Prusoff equation [48], which is used for calculation of Ki value is shown below.

(1) $$K_{i}=\frac{{\mathrm{IC}}_{50}}{1+\frac{\left[L\right]}{K_{d}}}$$

[L] represents concentration of radiolabeled tracer ([^3^H]CP55,940 in the current study) and K_d_ is the K_d_ value of the tracer. The Prism software was also used to determine the EC_50_ for the GTPγS binding assays.

For mRNA analysis, the change of IL-2 and TNF-α mRNA levels were analyzed by the ΔΔCt method with normalization to the housekeeping gene 36B4 to ensure equal amounts of mRNA loading. One-way ANOVA followed by Dunnett’s post hoc test was used to determine significant differences from the vehicle-treated group.

## 5. Conclusions

We demonstrated two ABK5-derived compounds, ABK5-1 and ABK5-2, are also CB_2−_subtype selective agonists, which have similar affinity to CB_2_ as ABK5, and no CB_1_ binding. These compounds also cause G-protein coupling. ABK5-1 and ABK5-2 have anti-inflammatory effects shown by inhibition of cytokine production and chemotaxis in Jurkat cells. Furthermore, ABK5-1 also inhibited pro-inflammatory cytokine production in BV-2 cells. These compounds may have stronger anti-inflammatory effects than ABK5, and can be potential drug candidates for treating inflammation.

## Figures and Tables

**Table 1:** Binding and G-protein-stimulation properties of ABK5 derivatives.

	CP55,940		GTPγS
	K_i_ (nM) ^a^		EC_50_ (nM) ^b^
Compound	CB_2_	CB_1_	CB_2_
ABK5-1	28 ± 5	N.B. ^c^	11 ± 14
ABK5-2	69 ± 19	N.B. ^c^	24 ± 25
ABK5-5	N.D. ^d^	N.D. ^d^	N.D. ^d^
ABK5-6	14 ± 2	>1000	10 ± 5
ABK5 ^e^	16 ± 14	N.B. ^c^	4 ± 3

^a^ Ki values were determined from competition binding assays using [^3^H]CP55,940. ^b^ EC_50_ values were determined from stimulation of [^35^S]GTPγS binding. ^c^ N.B.: No specific binding detected up to 10 μM of test compounds. ^d^ N.D.: K_i_ or EC_50_ value was not determined. ^e^ Parameters of ABK5 were determined previously and are shown for comparison [23].

## Data Availability

The data presented in this study are available on request from the corresponding author.
